# Practical prototype for energy management system in smart microgrid considering uncertainties and energy theft

**DOI:** 10.1038/s41598-023-48011-w

**Published:** 2023-11-27

**Authors:** Mohammed A. Saeed, Bishoy E. Sedhom, Abdelrahman S. Elbaghdadi, Ahmed W. Fadl, Mohammed S. Abdelwahab, Menna H. Elden, Bilal Naji Alhasnawi, Abdelfattah A. Eladl

**Affiliations:** 1 Electrical Engineering Department, Faculty of Engineering, Mansoura University, El-Mansoura, 35516 Egypt; 2 Communications Engineering Department, Mansoura Higher Institute of Engineering and Technology, El-Mansoura, Egypt; 3 Department of Electricity Techniques, Al-Samawah Technical Institute, Al-Furat Al-Awsat Technical University, Kufa, Iraq

**Keywords:** Renewable energy, Electrical and electronic engineering

## Abstract

The conventional electrical grid faces significant issues, which this paper aims to address one of most of them using a proposed prototype of a smart microgrid energy management system. In addition to relying too heavily on fossil fuels, electricity theft is another great issue. The proposed energy management system can simultaneously detect electricity theft and implement demand response tactics by employing time-of-use pricing principles and comparing real electricity consumption with grid data. The system uses the Al-Biruni earth radius (BER) optimization algorithm to make smart choices about how to distribute the load, intending to reduce energy consumption and costs without sacrificing comfort. As a bonus, it considers limitations imposed by battery charging/discharging and decentralized power generation. Incorporating sensors and SCADA-based monitoring, the system provides accurate measurement and management of energy usage through load monitoring and control. An intuitive mobile app also helps consumers connect, allowing for more active participation and better control over energy use. Extensive field testing of the prototype shows that by moving loads from peak period to another off-peak period, electricity expenditures can be reduced by up to 48.45%. The energy theft value was calculated to be 1199 W, proving that the system's theft detection model was effective.

## Introduction

**Figure 1:**
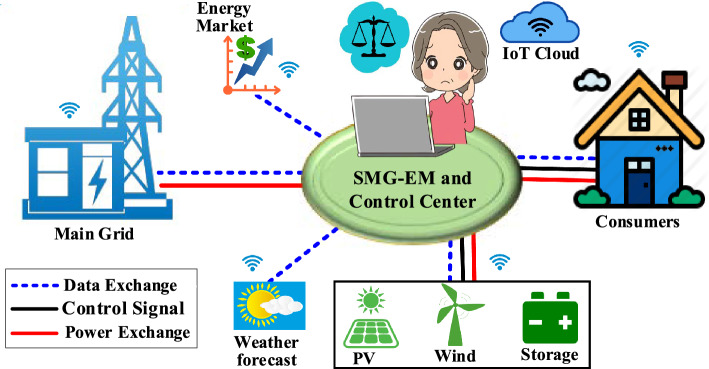
Typical schematic diagram for smart microgrids^4^.

Smart microgrids (SMGs) are small, localized power grids that can work alone or alongside the main grid. A blend of renewable energy sources, energy storage, and smart control systems optimizes resource utilization and responds to demand and supply changes in real-time^1^. SMGs can improve the resilience and stability of the power supply, reduce fossil fuel use, and lower energy costs. Figure 1 depicts a typical SMG schematic diagram. Power usage and production of the microgrid are monitored and communicated using smart meters which can detect abnormalities in usage patterns, such as spikes or drops, which are signs of energy theft. To prevent hacking and other threats, SMGs need strong cybersecurity like any other digital technology^2^. Smart microgrids use modern control systems and algorithms to optimize the use of existing resources and respond to demand and supply changes in real-time^3^. SMGs have the following characteristics,

- *Demand response management:* SMGs can use advanced algorithms to adjust the power consumption of connected devices in response to changes in demand, helping to balance the supply and demand of power.
- *Energy storage management:* SMGs can use energy storage systems to store excess energy generated by renewable sources, and release it as needed to meet demand.
- *Distributed energy resource management:* SMGs can use advanced algorithms to optimize the operation of distributed energy resources, to ensure the most efficient use of available resources.
- *Power quality management:* SMGs can use advanced control systems to maintain a stable and reliable power supply, even during disturbances on the grid.
- *Load balancing:* SMGs use advanced algorithms to balance the load across different distributed energy resources and energy storage systems to ensure a stable, reliable power supply.
- *Real-time monitoring:* SMGs use sensors and monitoring systems to collect real-time data on the status of the grid, allowing operators to make informed decisions about how to manage the system.

Energy theft threatens the economic viability and sustainability of smart microgrids. Theft of energy includes tampering, bypassing, and unlawful connections. Energy theft, including smart microgrids, costs the global energy industry billions of dollars. The dispersed architecture and distributed energy supplies of smart microgrids make them more vulnerable to electricity theft than conventional power grids^5^. Smart microgrids can analyze sensor and meter data to identify trends of energy theft.

There are many strategies for energy management systems for smart microgrids such as load management, generation management, and energy storage management^4^.

The control system of a microgrid must continuously analyze and prioritize loads to maintain a balance between power generation and consumption. Microgrid loads are usually critical or non-critical^6^. Critical loads in hospitals, nursing homes, and data centers are essential to running a facility and must never be interrupted. While discretionary loads can be temporarily cut off during peak times, emergency load sheds are needed to prevent blackouts. To reduce demand, heating, ventilation, and air conditioning systems, washing machines, and water heaters can be turned off during peak consumption. To prevent microgrid power disruptions, emergency loads in residential and commercial buildings must be shed. The authors of^7^ suggested using an intelligent load shedder module to isolate non-essential loads from the microgrid. They looked into ways to determine the priority and management of loads in different situations to improve efficiency. The classification, characterization, and tracking of loads necessitate systems for aligning loads with generation. Abdelsalam et al.^8^ suggested a hybrid demand-side management-multi-agent system energy management strategy for residential, commercial, and industrial microgrids. In^9^ the authors used the idea of electric springs in series to manage bus voltage and fault ride-through. They researched how to conserve electricity during situations of generating intermittent by reducing non-essential loads and maintaining critical loads. The authors of^10^ suggested using a combination of neural networks and fuzzy logic to create a system for managing energy demand, considering the significance of different types of loads, like residential, commercial, industrial, and hospital areas in an integrated energy management system (EMS). Additionally, they acknowledged that loads could also play a role in managing energy production in modern electrical systems.

Since distributed resources comprise the bulk of SMG systems, they necessitate complex power control strategies to function normally. The benefits of generation control in hybrid SMGs and the grid integration of diverse distributed generation (DG) were presented by^11^. Since solid oxide fuel cells (SOFC) are more efficient, researchers suggested employing them as supplementary units in the SMG. The authors in^12^ put forth two distinct approaches, known as frequency analysis and the electrical model of the triple combined cycle, to address the power variations caused by utilizing large-scale PV modules in a microgrid. The triple combined cycle (TCC) based SOFC model incorporates both gas and steam turbines. They utilized the swing equation for the grid-connected frequency model of the SOFC-TCC and examined the power response characteristics in the electricity model. An optimum microgrid framework that parallelizes capacitive-coupling inverters (CCIs) and inductive-coupling inverters (ICIs) is presented in^13^. It offers adaptive power sharing for CCIs using lower DC-link voltages to reduce power and losses. Using a neural network-based control layer, simulations, and experiments show accurate and fast sharing. Lin et al.^14^ explores time-domain analysis for grid-connected inverters (GCIs) in asymmetrical grid situations. A phase-locked loop-based harmonic state space model is created. Eigenvalue analysis and simulations show how grid impedance asymmetry affects GCI stability, underlining the need for weak grid context-specific control techniques. Experimental validation proves the model's efficacy and theoretical findings. The Robust antlion optimizer algorithm tracks maximum power point in solar PV systems^15^. Charge controller design, DC-to-AC converter tuning, and power scheduling minimize operational costs and stabilize the grid in the study. The study helps stakeholders find sustainable and affordable energy alternatives. Bi-level planning for a cooling-heat-electric multi-energy coupled distribution network is presented in^16^. It increases PV consumption with a distributed hydrogen-thermal storage system through a multi-objective expansion planning model for network dependability.

Energy storage systems (ESS) are essential for microgrid systems because they store and distribute electrical power to stabilize load and renewable energy generation, improve power quality, and ensure system reliability. ESSs are classified by storage and response as electrical, mechanical, chemical, electrochemical, or thermal.

Using microgrids, management of energy storage devices like batteries and flywheels in SMGs. Optimization of stored energy improves microgrid efficiency and dependability^17^. They can balance energy supply and demand, smooth renewable energy generating swings, and provide backup power during outages. Advanced control algorithms and communication systems are two of the technologies employed in SMGs to manage energy storage. Real-time monitoring and control of ESSs in microgrids can be enabled by integrating smart meters and other monitoring and control devices. The authors in^18^ proposed an idea for a mixed-mode EMS that can efficiently manage a microgrid by utilizing low-cost energy sources and determining the best energy storage option from an economic standpoint. This approach utilizes three distinct techniques: linear programming (LP) to handle economic power-sharing and continuous operation and LP and mixed integer linear programming (MILP) methods to handle the on/off utility mode operation. The particle swarm optimization method is used to compute the optimal positioning and size of ESS devices in order to minimize operational costs within the microgrid. However, if the depth of discharge of the battery is too high, the battery may degrade quickly and negatively impact the overall durability of the ESS. Tenfen et al.^19^ presented a MILP approach for optimal EMS using demand response (DR) and a penalty cost function, where the network investigated is a single-phase version of an AC network. However, the expense of battery degeneration owing to the increased depth of discharge is disregarded. The authors in^20^ addressed the issue of efficient battery energy storage and control in intelligent residential microgrid systems by designing a new adaptive dynamic programming algorithm. This algorithm uses a hybrid iterative approach with two iterations, known as the P-and V-iteration. The V-iteration minimizes cost within a given period, while the P-iteration improves the iterative value function. Through these iterations, the iterative value function is not guaranteed to increase monotonously and may converge to the optimal solution. In^21^, notable works on microgrid voltage regulation for supplying complex power loads have been highlighted, and then a filtration-based model predictive control method was implemented to evaluate the performance of ESS. The pinch analysis method was used in^22^ for conducting a comparative analysis of the ESS.

A droop control strategy was proposed in^23^, considering several factors such as the state of charge (SOC) speed balancing method, droop factor, voltage control, and current limit. The authors in^24^ improved controller gain with an interactive teaching–learning optimizer after proposing a robust fractional order controller for supercapacitor ESS to reduce control error and costs. Different control strategies of variable-speed pumped hydro storage were investigated in^25^ to determine the most effective approach at each level of the control hierarchy. The work in^26^ examined the efficiency during the under-voltage condition; and reported increased hydrogen production and considerable reductions in power fluctuations. Optimization approaches can increase the utilization of renewable energy sources, minimize the usage of fossil fuels, reduce energy expenditures, and improve the stability and reliability of the microgrid^27^.

This paper proposes a practical solution to improve the efficiency and security of energy management in smart microgrids. This paper presents a prototype of an intelligent microgrid energy management system. In order to optimize energy consumption and reduce costs, the system considers the uncertainty of renewable energy sources and the possibility of energy theft. The prototype employs machine learning algorithms and sensors to monitor and predict energy production and consumption and detect any unauthorized energy usage.

The main contributions of this article are concluded in the following points:

- The proposed method accounts for the Earth's radius, a crucial factor in accurately forecasting energy distribution across various locations. This consideration is necessary for optimizing the microgrid's load distribution to improve energy efficiency while also accounting for the unique geographical features of the area it serves.
- The incorporation of time-of-use pricing principles as part of the energy management system represents an innovative way to incentivize efficient energy consumption patterns among consumers.
- The use of the BER optimization algorithm in the proposed energy management system can lead to improved energy efficiency and cost savings.
- Developing an intuitive mobile app that empowers consumers to participate in and control their energy usage actively is a significant innovation. This enhances user engagement and promotes more sustainable energy practices.
- The paper's overarching novelty lies in its comprehensive approach to improving the dependability and sustainability of smart microgrid energy management. Addressing electricity theft issues and optimizing energy usage contributes to a more resilient and efficient energy infrastructure.

The rest of the article is organized into several sections: “Microgrid monitoring and control” section discusses the different tools for monitoring and controlling smart microgrids. “Problem formulation” section examines the problem formulation and the proposed energy management system for a smart microgrid. Al-Biruni Earth Radius optimization algorithm is explained in detail in “Al-Biruni earth radius (BER) optimization algorithm” section. “Practical implementation” section covers the practical implementation of the proposed system and the full results analysis. The article concludes with a summary of the findings in “Conclusion”.

## Microgrid monitoring and control

The monitoring system constantly analyzes its current state and makes necessary adjustments to guarantee the SMG equipment always functions smoothly and safely. The combination of software and hardware in this SMG guarantees the system's continuous, real-time operation.

### Internet of Things (IoT)

IoT provides real-time data and insights into the performance and operation of SMGs. This enables more efficient control of the microgrid and improves its performance and dependability. IoT monitors microgrids in several ways^28^:

- IoT devices can measure and track the amount of energy the SMG generates and consumes.
- IoT monitoring can detect and diagnose microgrid issues.
- IoT monitoring can improve grid stability and dependability by integrating renewable energy sources like solar and wind into SMGs, enhancing resilience.
- IoT devices enable remote monitoring and control of the SMG, especially in inaccessible regions.

IoT security solutions must consider real-time details, according to the authors of^29^. They looked at how IoT protocols might affect the time-sensitive needs of smart grid operations. The authors of^30^ built an IoT-based remote energy monitoring device for smart grid and household energy management, optimization, and conservation. The device efficiently tracks residential energy usage, reduces energy waste, and reveals energy behaviors. To standardize and unify structure parts, the authors of^31^ proposed an IoT-based EMS framework. Intelligent energy management and smart building modes allow for formulating relevant guidelines. A discussion of real-time microgrid monitoring was presented by^32^. The authors use web servers’ software, and data was collected wirelessly and transferred using an Arduino embedded system and ethernet network module. The authors of^4^ proposed an IoT-based SMG control and demand side management solution using genetic algorithm optimization. The control technique improves the voltage and frequency of the SMG during system load operations.

### Supervisory control and data acquisition (SCADA)

**Figure 2:**
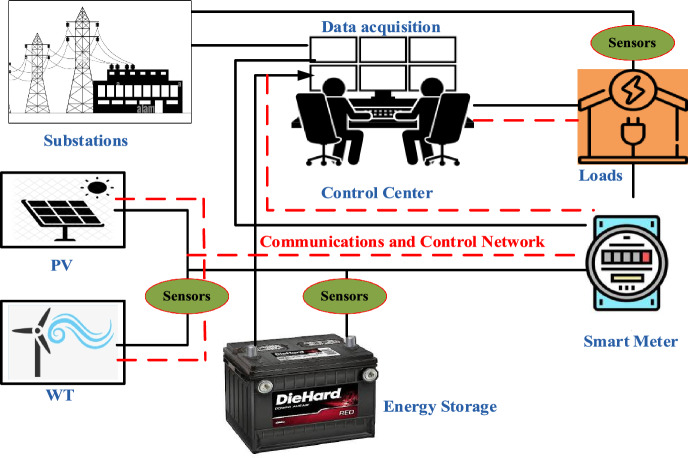
SCADA monitoring system for microgrids.

As shown in Fig. 2, the SCADA system can monitor and regulate SMG components such as generators, ESSs, and load control devices^33^. It can also collect microgrid performance data including energy generation and consumption and provide operators with real-time SMG status updates. This enables efficient and accurate SMG monitoring and control, ensuring maximal performance^34^.

SCADA systems can process and analyze massive volumes of data in real-time and perform complex control operations because they have more powerful processing capabilities than IoT devices^35^. Managing critical infrastructure and industrial systems is another SCADA benefit. SCADA systems monitor and control important infrastructure including power plants, water treatment facilities, and industrial systems like manufacturing and oil and gas facilities. SCADA provides the reliability and security these systems need. If equipment fails or other issues arise, SCADA systems frequently feature redundancy and fail-safe mechanisms to keep them running. IoT devices are cheaper and simpler and can be utilized for more general monitoring and control than SCADA systems. IoT devices are quicker to connect to the internet and accessible from anywhere^36^.

## Problem formulation

The proposed algorithm's primary goal is the minimization of the overall cost $({C}_{total})$, with the secondary goal being the electricity bill reduction. The second is to enhance user convenience in order to cut down on delay time or shift peak loads to off-peak times when power reserves are more plentiful. The cost reduction problem is defined as follows, where the deferrable loads are the flexible and movable needs:

1 $$Min.\Rightarrow {C}_{total}=\sum_{i=1}^{{N}_{app}}\sum_{t=0}^{T}({X}_{i}(t) \times {PR}_{i}(t) \times {EC}_{i}(t))$$

Subjected to the following constraints.

2 $$\left.\begin{array}{l}\begin{array}{l}{X}_{i}\left(t\right)= 0, if t\in {T}_{1}\\ {X}_{i}\left(t\right)= 1, if t\in {T}_{2}\\ 1 \le t\le T \end{array}\\ 1 \le i\le {N}_{app} \end{array}\right\}$$

where, ${X}_{i}\left(t\right)$ depicts the states that the appliances can be in as ON or OFF (1 = ON and 0 = OFF). ${PR}_{i}(t)$ indicates the cost of the electricity that the item $i$ has used during any time interval $t$. $T$ demonstrates the entire day, including peak and off-peak hours. Here, ${T}_{1}$ indicates the peak hours and ${T}_{2}$ describes the off-peak hours. $i$ denotes the appliances’ index number, and the variable specifies the number of appliances ${N}_{app}$. ${EC}_{i}(t)$ are the units of energy consumed by an appliance.

Minimal appliance wait time and optimal electricity cost are the goals of the design process behind user comfort $(UC)$. Customers should, therefore, always anticipate little expense and delay from utilities. Additionally, it aids in lowering customer irritation during times of high energy use. During this time, devices are assigned priorities, and the highest priority devices are given the first available slots in the off-peak schedule. It is possible to delay or even cancel low-priority device activities at peak times. As a result, unified communications are improved while device wait times are minimized. This leads us to the second goal, which may be stated as follows^4^:

3 $$Max.\Rightarrow UC= \left({E}_{app} + \frac{{E}_{CSaving}}{C}\right)$$

4 $${E}_{app}= \left(\alpha - \left(\frac{{D}_{app}}{24}\right)\right)$$

5 $${E}_{CSaving}= \beta \times \left(\frac{C}{100}\right)\times \left(\frac{Sc{h}_{cost}}{Max. cost}\right)$$

Subjected to the following constraints.

6 $$\left.\begin{array}{l}\begin{array}{l}0.3 \le \alpha , \beta \le 0.7\\ 1 \le delay \le 4\\ \alpha + \beta = 1\end{array}\\ \end{array}\right\}$$

where $\alpha$ and $\beta$ are the delay variables. ${D}_{app}$ is the maximum number of allowable delayed hours for the appliances per day. ${E}_{app}$ is appliance utility. ${E}_{CSaving}$ is the reduction in energy costs. $Sc{h}_{cost}$ is the cost of the non-deferrable appliances during the full day. $Max. cost$ is the cost of peak hours of the day.

**Figure 3:**
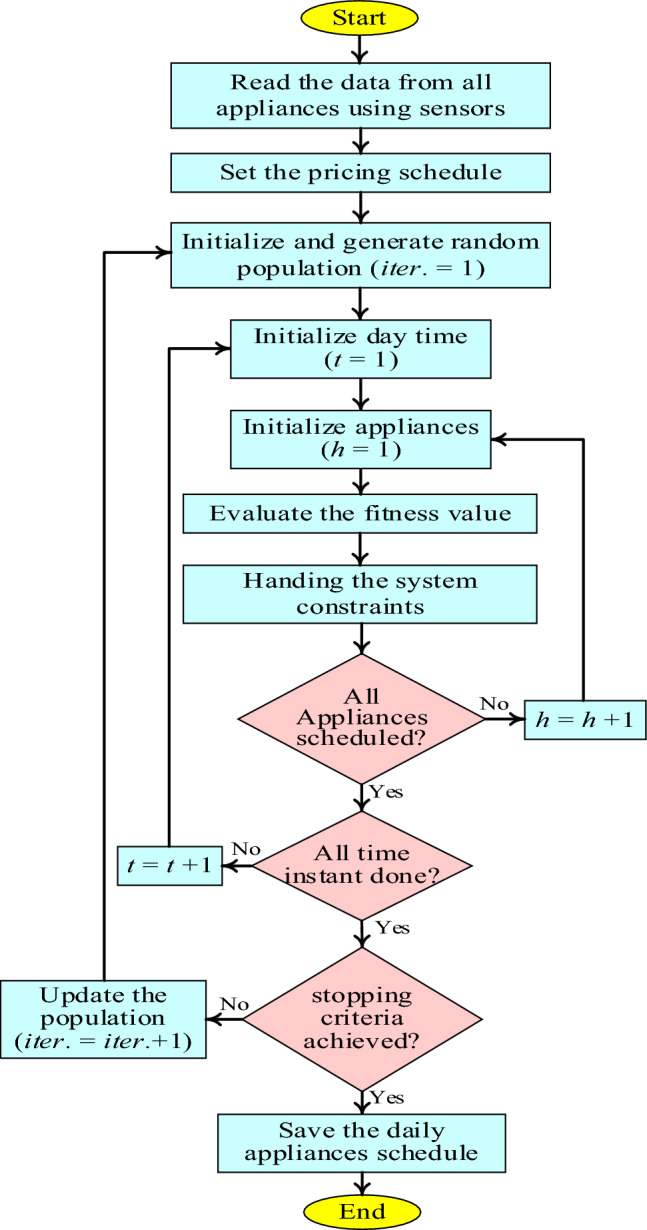
Flowchart of the proposed energy management algorithm.

As a rule of thumb, a 4-h window of delay per day for appliances is allowed. It's important to note that these 4 h were selected on purpose at peak times to draw attention to the longest device wait times. In the event of a power outage lasting longer than 4 h, the utility company is obligated to compensate its consumers by either refunding their money or reducing their electricity rates. An appropriate optimization method that addresses this issue is called BER optimization. Among its many benefits are its simplicity, global optimization, applicability to noisy settings, and scalability in terms of both the number of possible solutions and the amount of data needed to evaluate them. Additionally, it is probabilistic, solves effectively for mixed discrete/continuous problems, employs reward (objective function) information, and allows for multi-objective optimization. Figure 3 shows a diagrammatic representation of the proposed energy management algorithm.

## Al-Biruni earth radius (BER) optimization algorithm

**Figure 4:**
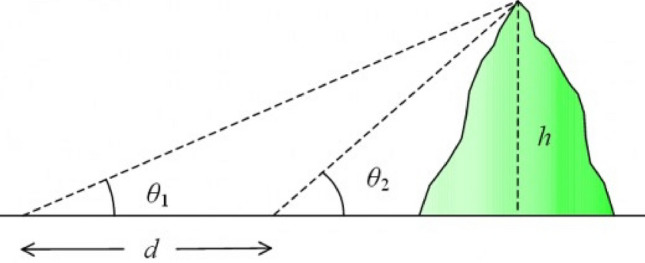
The Al-Biruni method for determining hill height.

BER optimization is a metaheuristic algorithm that is based on the premise of recreating the process of measuring the radius of the Earth utilizing the method devised by the Persian scholar Abu Rayhan Al-Biruni in the eleventh century. This approach was used to measure the radius of the Earth^37^. Recently, this optimization algorithm has been developed by^38^. Al-Biruni performed the calculation that determined the radius of the Earth in the eleventh century. He did this by standing on a hilltop and measuring the distance between the horizon and the ground. Al-Biruni took two separate readings. The first thing he did was measure the height of the peak. He took the angle to the mountain's crest twice, each time measuring it from a different vantage point. Using the following equation, which is depicted in Fig. 4, he was able to determine how high up the slope of the hill.

7 $$h= \frac{d\mathrm{tan}{\theta }_{1}\mathrm{tan}{\theta }_{2}}{\mathrm{tan}{\theta }_{2}- \mathrm{tan}{\theta }_{1}}$$

**Figure 5:**
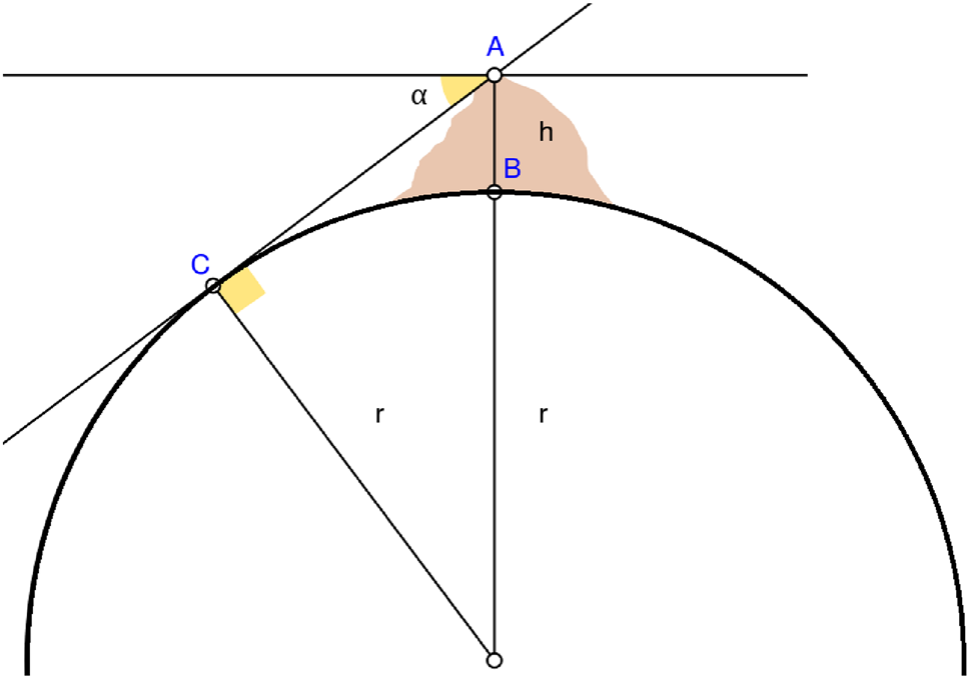
The Al-Biruni approach for Earth's radius calculation^38^.

Al-Biruni climbed to the top of the mountain and determined the angle of the horizon. The following equation, applied to his observations, allowed him to determine Earth's radius as depicted in Fig. 5.

8 $$R= \frac{h\mathrm{cos}\alpha }{1-\mathrm{cos}\alpha }$$

The cooperative optimization algorithm used Al-Biruni to explore and exploit, mimicking swarm intelligence. Wild swarms live together and share information. They take turns searching for food and avoiding predators. Members worked together in smaller groups and on their own. In swarms, ant and bee colonies cooperate. Each member's actions strengthen the swarm. The colony's workers feed the warriors. The fact that people are generally divided into smaller groups to focus on specific activities at specific times inspired BER. Exploration and exploitation are used to find the best optimization solution. Here, BER divides people into two groups, one for each pursuit. BER's exploitation and exploration stages exhaustively search the search space to prevent local optimums. Most cooperative optimization approaches need collaborative action after each iteration, which may maintain inferior solutions or converge to a local optimum. To avoid this, BER maintains a pool of agents that collectively search for a bigger search space over time. If BER's performance doesn't improve after three solution mutation iterations, search space exploration will expand significantly.

### Fundamental ideas

BER, an optimization algorithm, utilizes a vector representation to denote individuals in the population. The vector is represented as $\overrightarrow{S}=\left\{{S}_{1}, {S}_{2}, \dots , {S}_{d}\right\} \in {R}_{d}$, where d denotes the size of the search space, and ${S}_{i}$ represents a feature or parameter in the optimization problem. The algorithm evaluates the performance of each individual using a fitness function, f, to determine how well the individual performs up to a specific point. To find an optimal vector that maximizes the fitness function, BER performs an optimization procedure with several phases. The process begins with a set of random individuals, or solutions, to search through the population. For BER to commence the optimization process, it requires specific parameters such as the fitness function, lower and upper bounds for each solution, dimension, and population size.

### Exploration–exploitation balance

The proposed algorithm divides the population into subgroups to balance the tasks of exploitation and exploration. The number of individuals in each group is dynamically adjusted to optimize the fitness values. Initially, the population is divided into two groups: an exploration group and an exploitation group^39,40^. The exploration group comprises 70% of the population, while the exploitation group consists of 30% of the population. To improve the fitness values of individuals in each group, the number of individuals in the exploitation group is increased from 30 to 70% over the optimization iterations. Conversely, the number of individuals in the exploration group decreased from 70 to 30% over the iterations. This process enhances the global average of the fitness of individuals, promoting more significant improvements. To ensure convergence of the optimization process for the population, the elitism strategy is utilized, whereby the leading solution is kept if no better solution is found. If a solution's fitness does not improve significantly over three iterations, the algorithm applies a mutation operation to generate another exploration individual as it may be a local optimum.

### Exploration operation

Exploration involves both identifying promising regions in the search space and avoiding getting stuck in local optima by moving towards the best solution. To achieve this, individuals in the exploration group use a strategy of searching for potential areas around their current position in the search space. They do this by repeatedly searching for better options in terms of fitness value among the surrounding feasible alternatives. The BER optimization algorithm employs the following equations to guide this process.

9 $$r=h \frac{cos\left(x\right)}{1-\mathrm{cos}(x)}$$

10 $$\overrightarrow{D}= \overrightarrow{{r}_{1}}.\left(\overrightarrow{S}\left(t\right)-1\right)$$

11 $$\overrightarrow{S}\left(t+1\right)= \overrightarrow{S}\left(t\right)+ \overrightarrow{D}.\left(2\overrightarrow{{r}_{2}}-1\right)$$

where $0<x\le 180$, $h$ is a number that is chosen at random from the available options within the range [0, 2], $\overrightarrow{{r}_{1}}$ and $\overrightarrow{{r}_{2}}$ are vectors of coefficients whose values are determined by how they are measured by (9), $\overrightarrow{S}(t)$ is the solution vector at iteration $t$, and $\overrightarrow{D}$ is The diameter of the circle refers to the distance from the current position of the search agent to the farthest point it will search for potential areas. It determines the range of the search space that the agent will explore in its search for better solutions.

### Exploitation operation

The mission of the exploitation group is to enhance already implemented strategies. In each cycle, the BER determines each person's fitness level and picks out the top performer. As can be seen in the next sections, the BER uses two distinct methods to achieve exploitation.

#### Heading toward the best solution

The following equations are used to move the search agent toward the best solution:

12 $$\overrightarrow{S}\left(t+1\right)={r}^{2} (\overrightarrow{S}\left(t\right)+ \overrightarrow{D})$$

13 $$\overrightarrow{D}= \overrightarrow{{r}_{3}}\left(\overrightarrow{L}\left(t\right)-\overrightarrow{S}\left(t\right)\right)$$

where $\overrightarrow{{r}_{3}}$ is a random vector calculated using (9) that controls the movement steps toward the best solution, $\overrightarrow{S}(t)$ is the solution vector at iteration $t$, $\overrightarrow{L}$ is the best solution vector, and $\overrightarrow{D}$ refers to the distance vector.

#### Investigating the area around the solution

**Figure 6:**
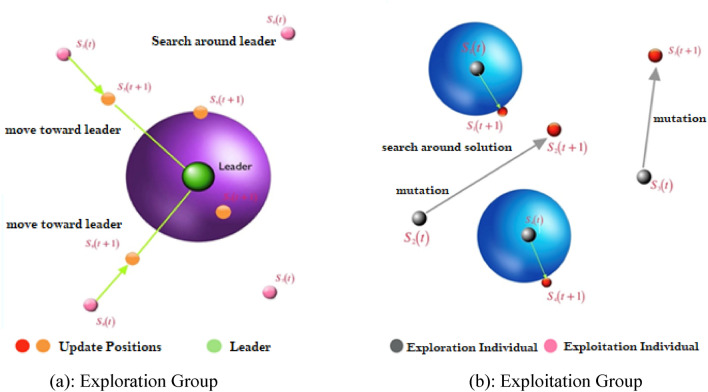
Demonstration of exploration and exploitation activities.

The area around the most promising leader is the most promising one. Therefore, some people look around locally for the greatest answer, hoping to unearth something better. The following equation is used by the BER to do this operation:

14 $$\overrightarrow{S}\left(t+1\right)=r \left(\overrightarrow{L}\left(t\right)+ z+ \frac{2 \times {t}^{2}}{{N}^{2}} \right)$$

where z is a random number in the range [0, 1], and N is the total number of iterations. Figure 6 is a visual representation of the exploration and exploitation processes.

### Mutation operation

The BER conducts research using a variety of methods, one of which is mutation. It is a genetic operator that is used to develop and maintain variation in population populations. It is possible to think of it as a probabilistic local random disruption of one or more components in people. It stops early convergence by assisting in the avoidance of local optima, and such a movement in the search field acts as a springboard to another interesting subject. The mutation is significant for the outstanding exploration possibilities offered by the BER.

15 $$\overrightarrow{S}\left(t+1\right)= \overrightarrow{k}\times {z}^{2} -h\frac{cos\left(x\right)}{1-\mathrm{cos}\left(x\right)}$$

### Best solution selection

The best solution is selected for the following iteration by the BER, guaranteeing the high quality of the solutions found. While the elitism approach improves algorithm performance, it may lead to unexpected behavior when used for multimodal functions. It's worth noting that the BER's mutation procedure and seeking around explorers in the group result in excellent exploring abilities. The BER's robust exploratory capabilities allow it to delay convergence.

## Practical implementation

**Figure 7:**
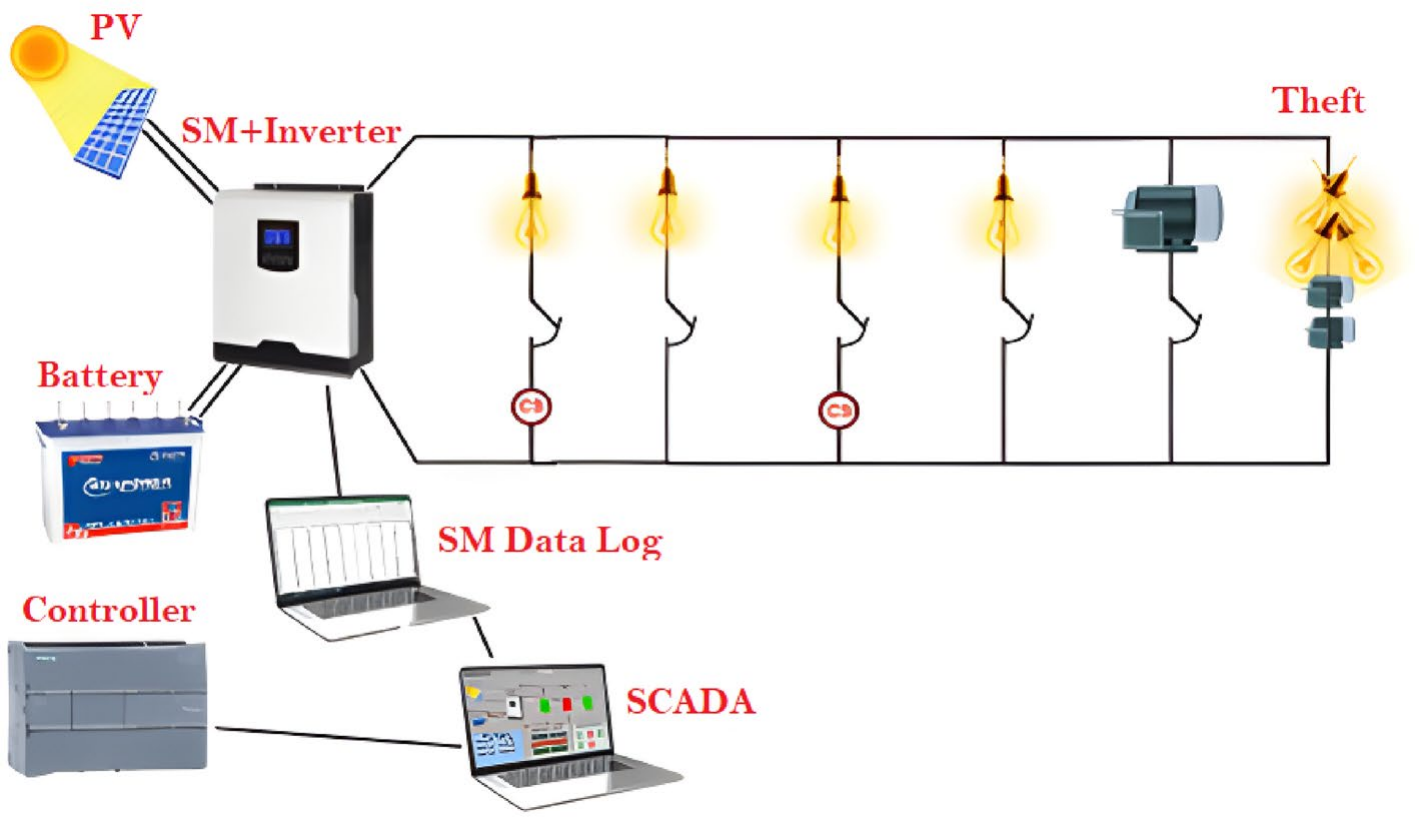
Schematic diagram of the practical test system.

During this part of the study, a working model of the SMG system is developed and tested in the laboratory. This prototype is made up of photovoltaic panels, a battery, a programmable logic controller (PLC), an analog input module, a smart meter, an inverter, AC loads, motors, lighting, relays, and current sensors. Figure 7 is a representation in the form of a schematic diagram of the prototype that has been implemented. The controller is equipped with a central processing unit, and it is responsible for the transmission and reception of output and input signals. It had digital outputs attached to it in the form of the relays, and it had analog inputs in the form of the load currents. The software for totally integrated automation (TIA) Portal was utilized in the process of designing the system control program. Ladder diagrams were used as the primary graphical representation.

**Figure 8:**
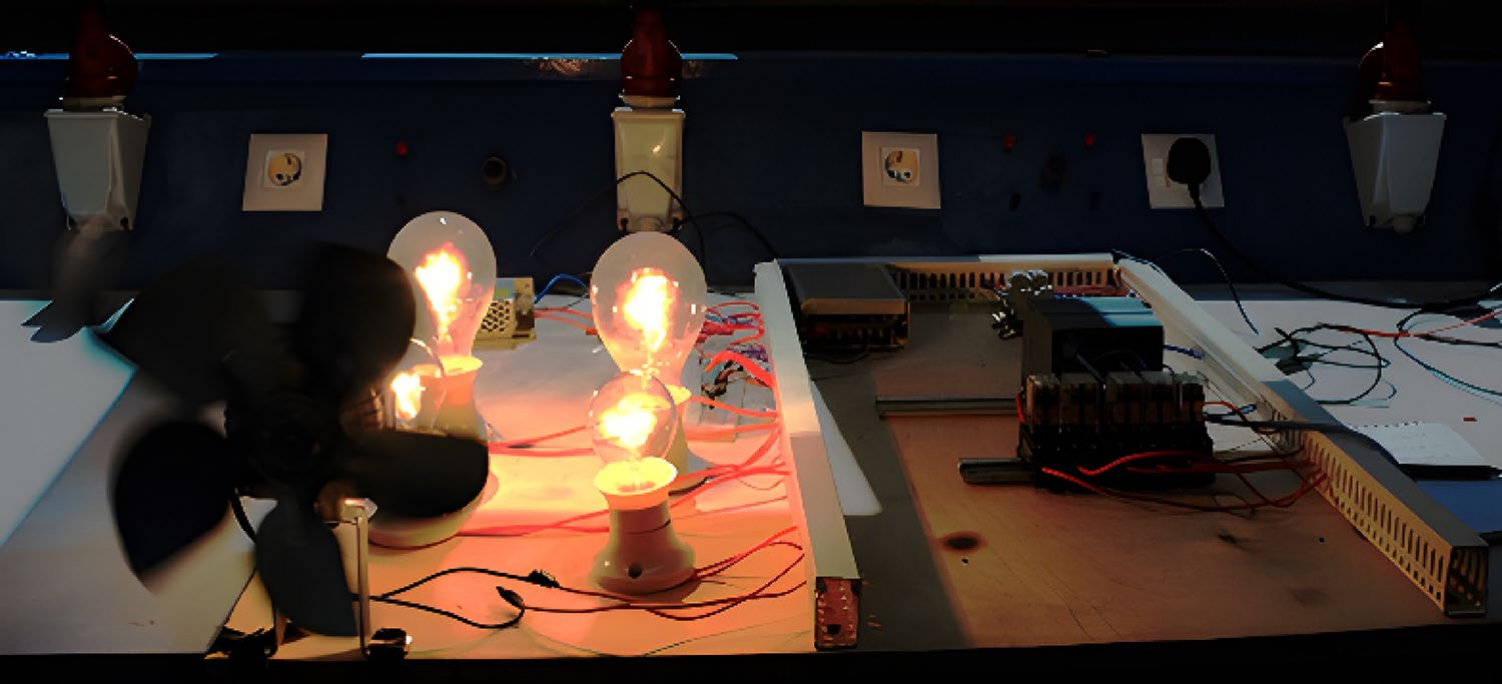
The practical test system prototype.

The value of the system loads that are measured from the sensors are included as one of the system inputs. Other examples of system inputs include the solar panel output value, the battery state of charge %, the inverter output for loads, and the value of the solar panel output. On the other hand, the signals sent to the relays, which are employed in the process of controlling the loads, are regarded as system outputs. Figure 8 is a representation of the actual test system prototype that will be used for the proposed EMS. The data regarding the PV input power, PV input voltage, output apparent power, output active power, battery voltage, and battery capacity are measured from the smart meter by way of a printer cable that is connected between the smart meter, inverter, and computers. The data are kept track of in an Excel sheet, and the control system is programmed to respond appropriately if there is a shift in the values that are being monitored at any given time of the day.

The PLC controller is linked to the rest of the system via an OPC-UA Server/Client, and the PLC and the Excel sheet serve as storage for the communication protocol known as dynamic data exchange protocol. The system's output consists of relay signals used to manage the loads in the system. Here's how the controller gets put to use.

i. The disconnection and reconnection of loads are governed by one of three distinct kinds of procedures. The first difference is that in the event of a shortcoming in the inverter settings, the sheddable loads will be disconnected until the issue has been resolved. The second strategy involves disengaging certain types of loads once a deficit is detected and then reconnecting them after a certain amount of time has elapsed, regardless of whether or not the deficit has reached the maximum allowable level. This category of load is referred to as shiftable load. Third, when there is a shortage, the quantity of electricity that is given to the load can be regulated until the issue is resolved. This can be done by managing the amount of electricity that is sent to the motors or the DC lamps; this category of load is referred to as a controllable load.
ii. The priority of loads at various points during the day is used to determine how the order of these operations is determined and regulated. When a shortfall is discovered at the start of the day, the shiftable load, the sheddable load, and the controlled load are separated. When a power shortage hits in the middle of the day, however, the procedures for disconnecting the lines are carried out in a different order. As an illustration, first controllable, then sheddable, and last shiftable. The priority level of the same load at various times throughout the day results in a variable level of urgency. For instance, the lamps that are active during the afternoon period do not have a priority, which means that they can be moved during the first action. On the other hand, the identical lamps that are active during the night period have a high priority and therefore cannot be moved during the first action.
iii. The price is set to be higher during peak hours and lower during off-peak hours.
iv. Depreciated costs are calculated by multiplying the sensor readings by the current market price.

**Figure 9:**
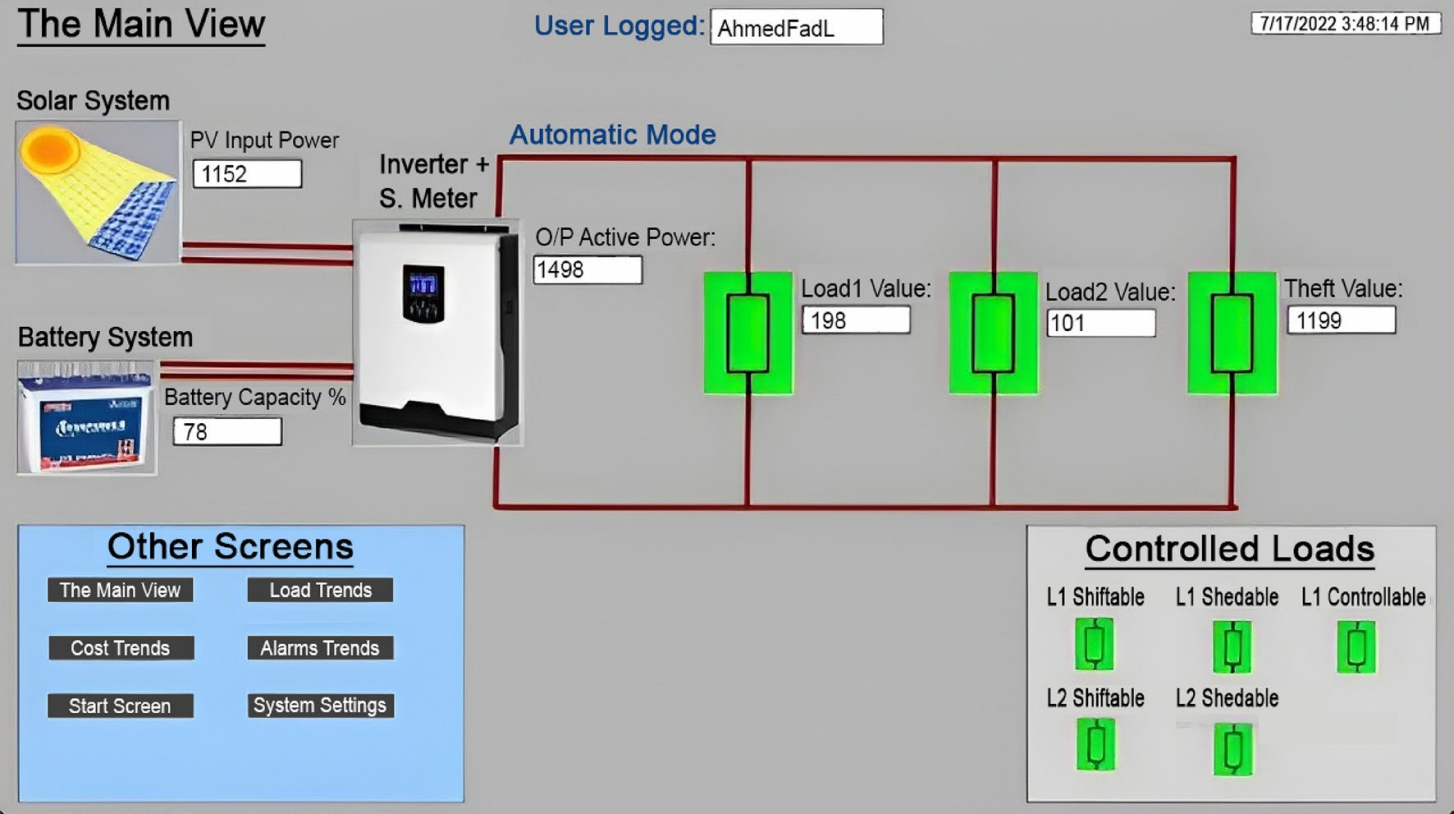
Main view of SCADA system.

**Figure 10:**
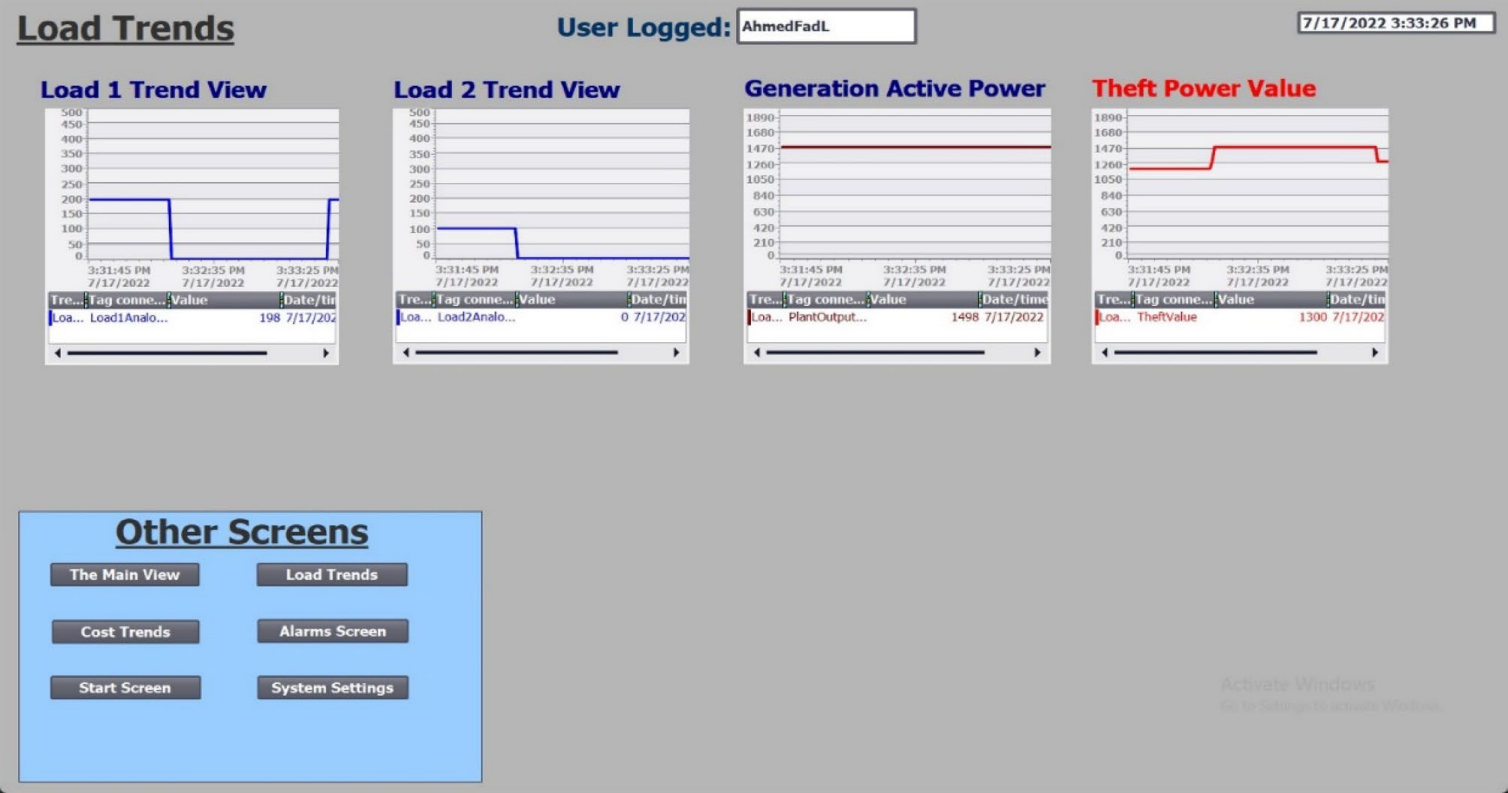
Load trends from SCADA screen.

For the purpose of designing the control system, TIA portal software is utilized. WinCC software was used to create SCADA systems, and these systems were then linked to the PLC control system. The processes of disconnecting and reconnecting loads can either be handled automatically, as was explained earlier, or manually, by clicking buttons on the screen, as illustrated in Fig. 9. In addition to the values of the stolen electricity that are displayed in Fig. 10, the consumptions of the loads that were taken from the sensors were displayed approximately every hour.

**Figure 11:**
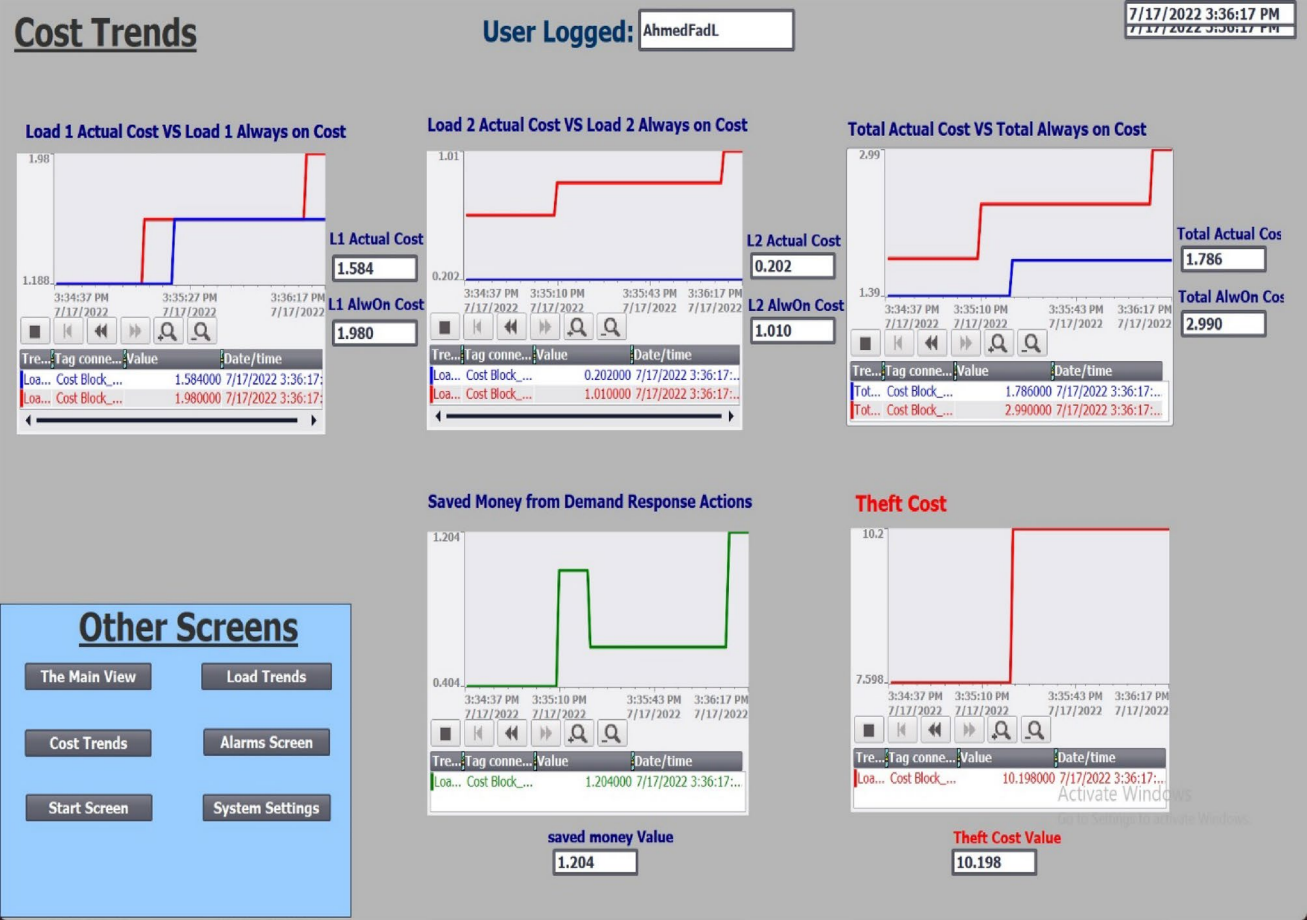
Cost trend from SCADA screen.

**Figure 12:**
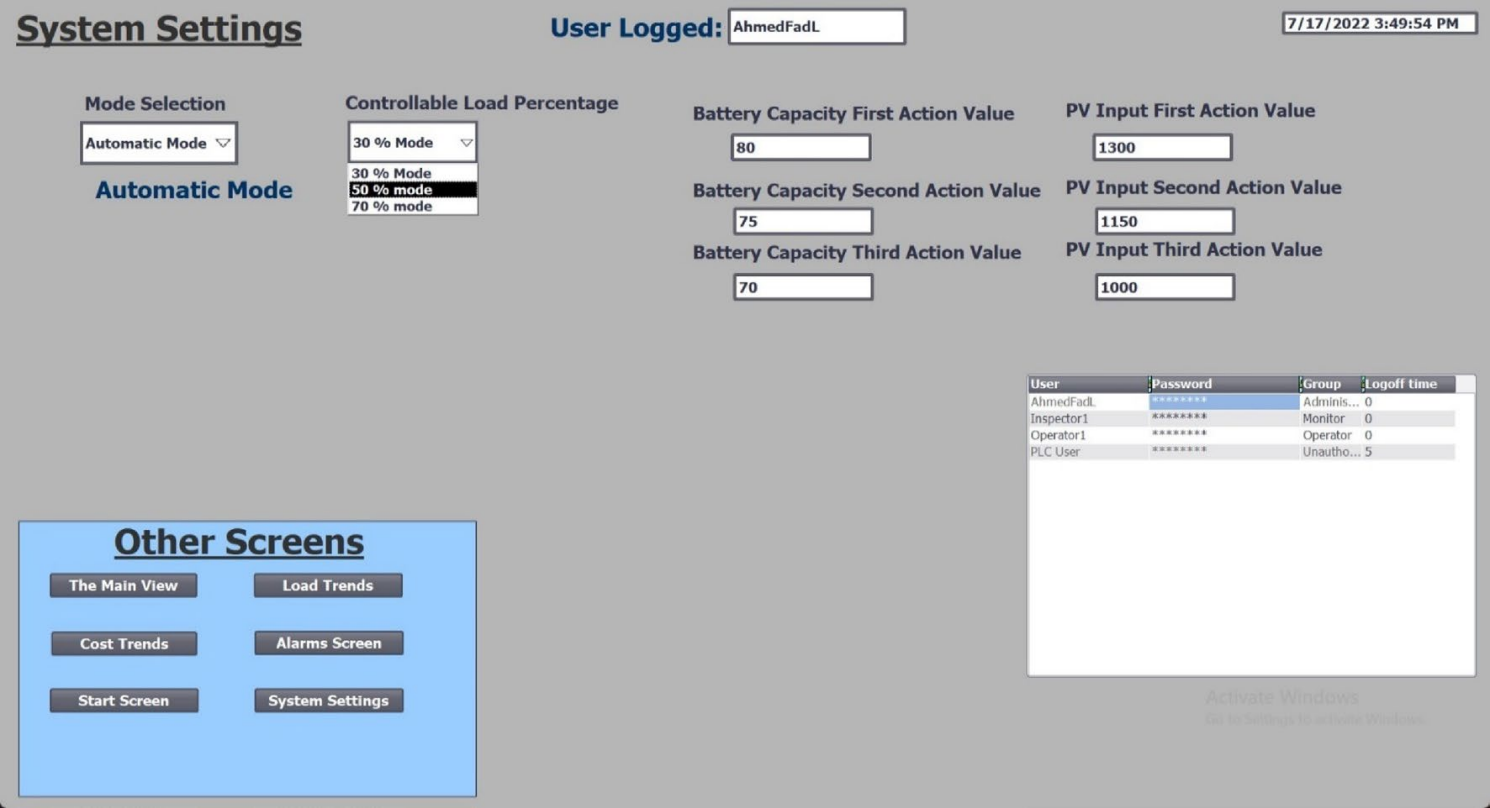
System setting, the stages of disability based on the time of the day, battery capacity, and PV input power.

**Figure 13:**
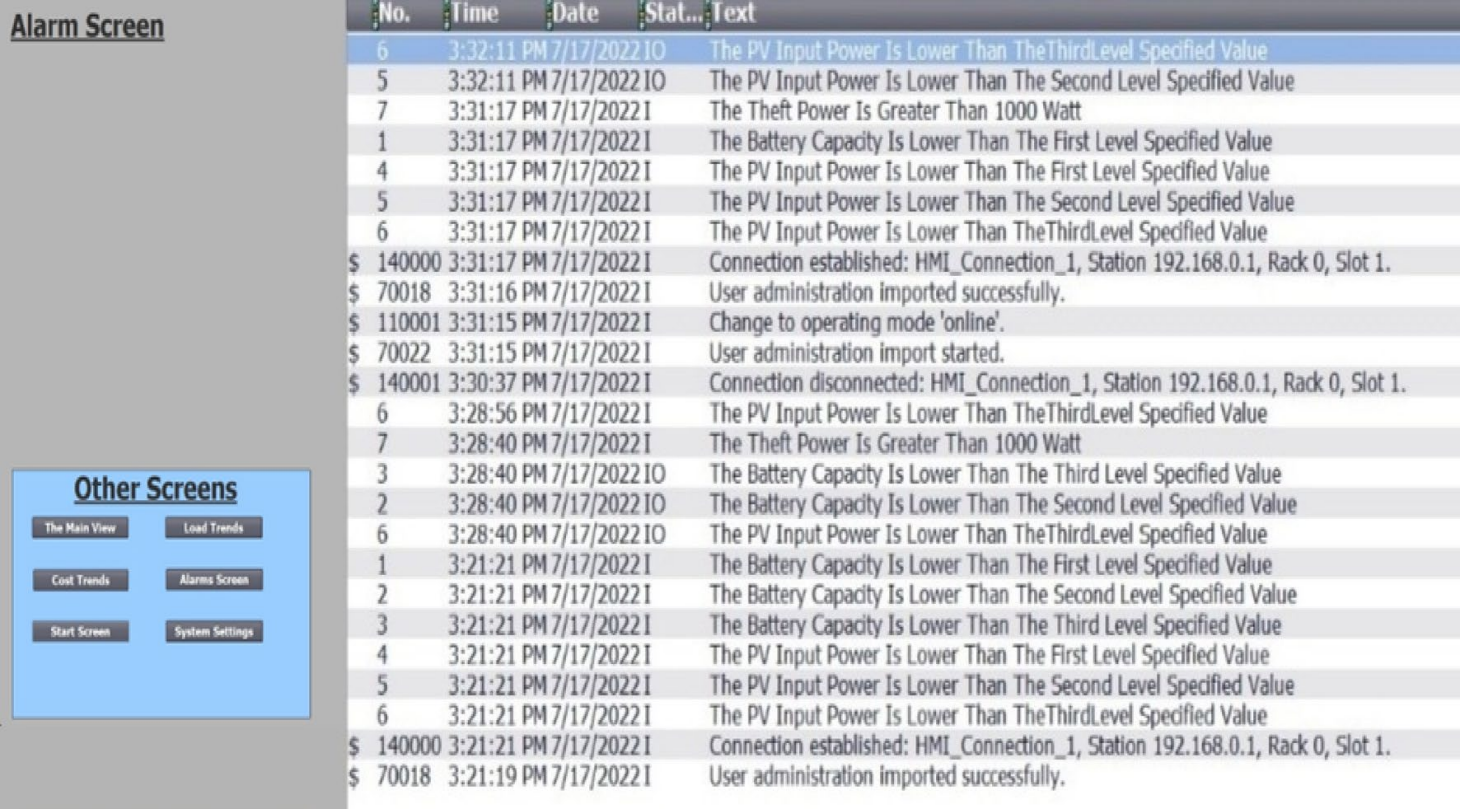
Alarms from SCADA screen.

The prices of the electricity that was taken were also retrieved, as shown in Fig. 11. The prices of the electricity that was stolen were displayed, as well as the prices that were available from the operations of disconnection and connection that were carried out by the control room. Controlling the value of the degrees of incapacity at which the control action is taken, and the deferrable load have each been incorporated into a settings page for the station. The system can be controlled manually or automatically depending on whether the current username has the authority to do so or not. Manage the login names and passwords for everyone who is authorized to enter the station, as well as their capabilities and the aspects of their environment that they are permitted to alter, as shown in Fig. 12. Figure 13 provides an illustration of the alerts and warnings page that has been created for the station.

**Figure 14:**
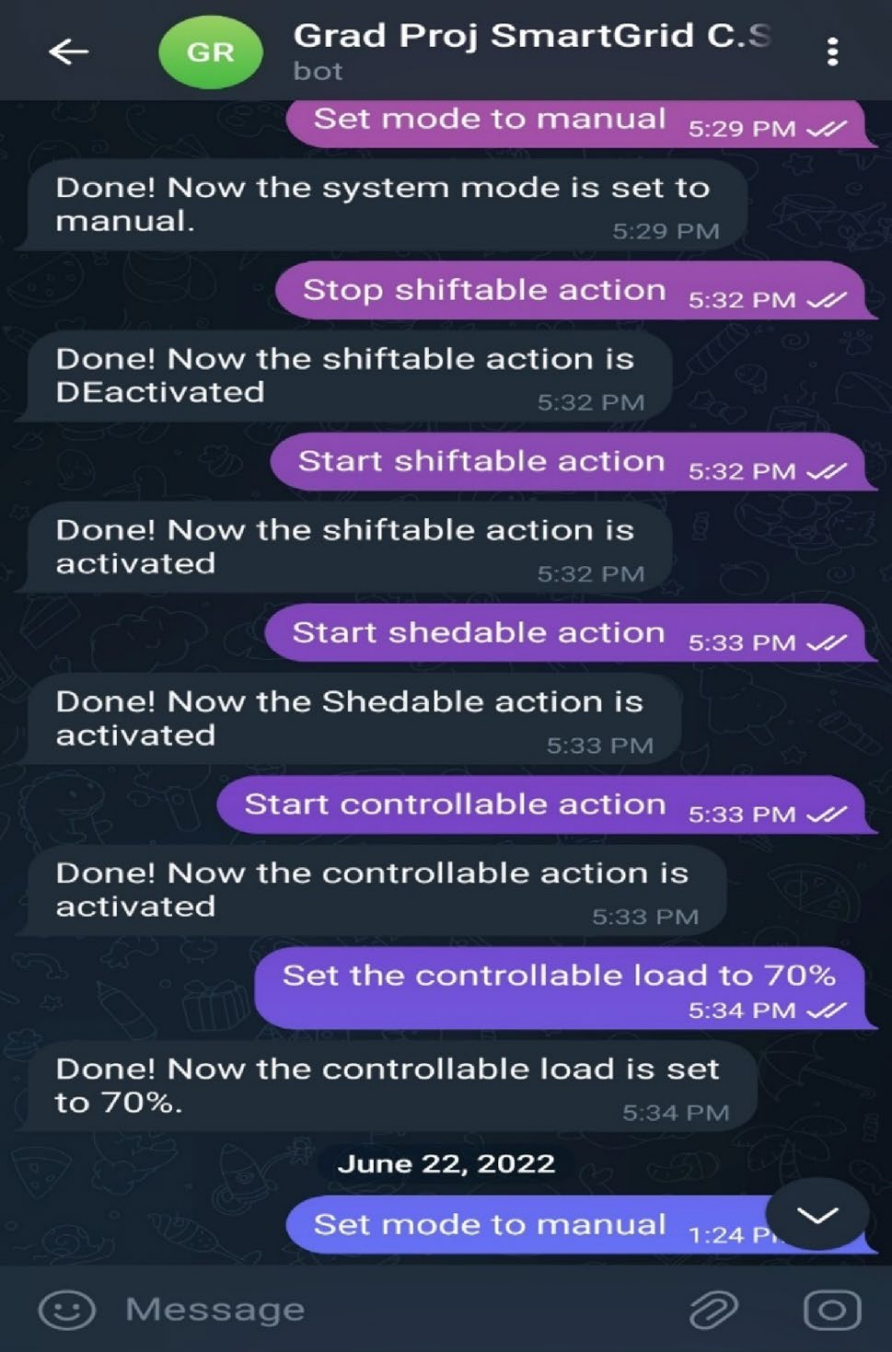
Performed Telegram Bot program.

Through the combination of IOT and Telegram, the Telegram Bot may be able to perform the same tasks as SCADA on a mobile phone. The Telegram Bot and the control system, which was programmed on the red node platform, were able to connect with one another thanks to the OPC-UA Server/Client communication protocol. The Bot is able to issue commands to the system as well as query about the settings that are currently in place. A real-world application of the Telegram Bot commands is demonstrated in Fig. 14. The following is a list of the commands and queries that can be carried out with the help of the Telegram Bot:

- Change the operating mode by typing 'Set mode to manual' or 'Set mode to automatic'.
  - If you are in manual mode, you can start the DR actions by typing 'Start shedable action' or 'Start shiftable action' or 'Start controllable action'.
  - Also, you can stop these actions by replacing the 'Start' word with the 'Stop' word.
  - Concerning the controllable load, if you are in manual or automatic mode, you can change its percentage by typing 'Set the controllable load to 30%' or 50%, or 70%.
- You will not only take action, but you will also get the current readings of the system like:
  - Knowing the current Battery System Capacity Percentage value by typing 'What is the battery capacity percentage?'.
  - Knowing the current PV System Input Power value by typing 'What is the PV input power?'.
  - Knowing the current Plant Output Active Power value by typing 'What is the plant output active power?'.
  - Knowing the current Theft Power value by typing 'What is the theft value?'.
  - Knowing the current Theft Cost value by typing 'What is the theft cost value?'.
  - Knowing the current Total Load Cost value by typing 'What is the total load cost value?'.

## Conclusion

This paper introduced a novel approach to energy management by combining demand response with the ToU pricing model. With the help of the Al-Biruni Earth Radius optimization method, it was conceivable to strike a balance between minimizing energy consumption and maximizing human comfort. To further fortify the smart microgrid's safety, a theft detection device that tracks the gap between electricity withdrawal and consumption has been implemented. The proposed system also included the management of inverter and smart meter-connected loads, allowing for flexible responses to power outages. Daily demand trends were used to determine the order of priority for load shedding, and management systems were developed to shed, shift, or control loads as needed. SCADA solution, which included the WinCC software and an IoT Telegram Bot, also allowed for effective monitoring and control, even from far away. Many promising avenues for further study and advancement have been identified as a result of this paper. Advanced machine learning algorithms could be used to improve the theft detection mechanism; further optimist the BER algorithm; investigate strategies for dynamic load management; incorporate energy storage solutions; bolster cybersecurity measures; encourage user participation; evaluate environmental impact; explore grid expansion and interconnection; and so on. A more sustainable and resilient energy future can be achieved by addressing these issues, which will contribute to the ongoing development of smart microgrid energy management.

## Data Availability

The datasets used and/or analyzed during the current study are available from the corresponding author on request.

## List of symbols

$${C}_{total}$$: Overall cost
${N}_{app}$: Number of appliances
$T$: Duration of the day
$$t$$: Time interval
$$i$$: Appliance number
${X}_{i}(t)$: Appliances ON/OFF states
${PR}_{i}(t)$: Cost of the electricity consumption
${EC}_{i}(t)$: Appliance energy consumption cost
$${T}_{1}$$: Peak hours
${T}_{2}$: Off-peak hours
$$UC$$: User comfort
$${E}_{app}$$: Appliance energy
${E}_{CSaving}$: Energy costs reduction
$$\alpha$$,$$\beta$$: Delay variables
${D}_{app}$: Maximum allowable delayed hours for appliance per day
$Sc{h}_{cost}$: Cost of non-deferrable appliance per day
$Max. cost$: Cost of peak hours
$h$: Height above the earth's surface
$d$: Horizontal distance from the observer to the base of the hill
${\theta }_{1}, {\theta }_{2}$: Angles measured from the observer's position to two points on the hill
$$R$$: Earth's radius
$\alpha$: The angle of depression from the horizontal
$$\overrightarrow{D}$$: Distance vector
$\overrightarrow{{r}_{1}}$, $\overrightarrow{{r}_{2}}$,$\overrightarrow{{r}_{3}}$: Random coefficients vectors
$\overrightarrow{S}\left(t\right)$: Solution vector at iteration t
$\overrightarrow{S}\left(t+1\right)$: Solution vector at iteration (t + 1)
$\overrightarrow{L}\left(t\right)$: Best solution vector
$z$: Random number in the range [0, 1]
$N$: Total number of iterations
*BER*: Al-Biruni earth radius
*SMGs*: Smart microgrids
*EMS*: Energy management system
*DG*: Distributed generation
*SOFC*: Solid oxide fuel cells
*TCC*: Triple combined cycle
*CCIs*: Capacitive-coupling inverters
*ICIs*: Inductive-coupling inverters
*GCIs*: Grid-connected inverters
*ESS*: Energy storage systems
*LP*: Linear programming
*MILP*: Mixed integer linear programming
*DR*: Demand response
*SOC*: State of charge
*IOT*: Internet of Things
*SCADA*: Supervisory control and data acquisition
*PLC*: Programable logic controller
*TIA*: Totally integrated automation
